# Diseases of the Blood

**Published:** 1905-02-25

**Authors:** 


### Progress in Medicine and Surgery.

### DISEASES OF THE BLOOD.

**Rôle of the Lymphocyte.**—At the meeting of the British Medical Association at Oxford a discussion on the rôle of the lymphocyte was opened by Gulland,[1] who said that four varieties of non-granular cells were generally recognised—large and small lymphocytes, large mononuclear and transitional—but that he was of opinion that all these were merely stages in the development of one type, and in no way related to the polymorphonuclears. In all the cytoplasm was faintly basophile, gradations in depth of colour depending on the thickness and arrangement of the strands and nodes of the cytoplastic reticulum. Differences in the shape and position of the nucleus depended on the pull exerted by radii diverging from the centrosome; the nucleus being first pushed to one side and then deformed. He therefore classed the leucocytes into lymphocytes on the one hand, and neutrophiles, eosinophiles and basophiles, or mast-cells, on the other. In adult age these two groups of cells were distinct and reacted differently to stimuli—the first were found normally in the blood, the second in the marrow. The function of the lymphocytes was probably largely protective, and their chief habitat, outside the lymph glands and marrow, was in the lymphatic tissue round the hollow tubes of the body—alimentary and expiratory tracts—spots occupied by saprophytic, non-virulent or attenuated organisms. Muir said that the hyaline wandering cell with oval nucleus might be taken as the primitive type of white bloodcell, next he would place the coarsely granular eosinophile, while the cells with well-marked neutrophiles did not occur until the fifth month of intrauterine life. Non-granular cells in large numbers were chiefly associated with subacute and chronic conditions eosinophiles were attracted by special chemotactic substances in relatively non-acute conditions, while the finely granular cells were chiefly concerned in acute bacterial affections. Beattie said that at the site of irritation in early inflammatory processes various kinds of cells accumulated, many of which had migrated from distant parts, others produced locally from proliferation of endothelial cells and fixed connective cells. In acute inflammatory exudations transition stages between the lymphocytes and large mononuclears were numerous. Transitional cells were but few in the blood, probably owing to the fact that they were formed in lymphoid tissue outside the vessels, and only a few were washed in by the overflow from lymphatic channels, or possibly by migration. The larger forms of lymphoid cells were undoubtedly phagocytic. Drysdale estimated that there were about 7,500 leucocytes per cubic millimètre of the blood, made up of 5,000 polymorphonuclears, 2,000 lymphocytes, 350 large mononuclears, and 150 eosinophiles. All differential counts should be stated in absolute numbers per cubic millimètre, for if this is not done, a decrease in the number of any one class of cell leads to an increase—which is really only an apparent one—in the other classes. Lymphocytosis when it did occur afforded no conclusive information, and in the majority of cases it was at present impossible to explain the variations in their number. MacCullum said that examination of the blood during the height of typhoid fever showed a definite leukopenia, due to a decrease in the polymorphonuclear leucocytes, whilst after an initial fall the relative and absolute numbers of lymphocytes were greatly increased. Ferguson said that in variola an excessive production of cells of the lymphocyte classes took place, which were found in very high proportions in the blood and in the skin. In the skin they formed a notable constituent of the exudation in the earlier stages of the eruption. These changes were accompanied by considerable activity of the lymphoid tissues. Houston was of opinion that all the cells of the lymphocytic group were formed in lymphoid tissue. The larger forms of mononuclear cells were undoubtedly amœboid and phagocytic, and even the smaller forms were motile. Sepsis in the child produced a leucocytosis which was mainly a lymphocytosis; in the adult one which was mainly a polymorphonuclear leucocytosis. Similarly, leukæmia in the child is generally a lymphocytosis; in the adult, a leucocytosis characterised by an increase in the granular cells.

**The Glycogen Reaction in Blood** is the subject of a paper by Gulland,[2] who says that the solution which serves for fixing, staining and mounting, is composed of iodine 1 gram, iodide of potash 3 grams, distilled water 100 c.cm., with enough gum acacia to make the fluid of a syrupy consistence. A drop of this solution is placed on a clean slide, and an ordinary air-dried blood-film on a cover glass lowered on to it, allowed to remain half a minute, and then displaced towards one end of the slide, or the surplus fluid removed by filter paper. The gum soon sets and the preparation will keep for some weeks. It should be examined with an oil-immersion lens. If no glycogen is present none of the leucocytes will show the faintest test of red or brown; if the reaction is positive the change occurs in the polymorphs only; lymphocytes and eosinophiles are never affected. The cells may give the brown or reddish brown glycogen reaction as a diffuse coloration, as fine granules scattered through the cell body, or as coarser granules mostly found at or near the periphery. The nuclei are not affected. The granules are parablastic and imbedded in the reticular substance. The nature of the staining substance is probably a loose combination of glycogen with a proteid. The number of cells affected varies greatly, from 2 or 3 per cent. to nearly all. Clinically the following are the chief conditions in which a positive reaction is constant—croupous pneumonia, broncho-pneumonia, empyema (not in serous effusions), abscess and gangrene of the lung, septicæmias and pyæmias of all kinds (*e.g.*, ulcerative endocarditis and puerperal septicæmia), all advancing suppurative conditions unless there be absolutely free drainage, septic gangrene of all kinds, appendicitis and peritonitis, late typhoid, advanced phthisis, miliary tuberculosis, gonorrhœal arthritis. The reaction is negative in rheumatic fever, chronic rheumatoid arthritis, chorea, rickets, catarrhal jaundice, dry and serous pleurisy, bronchitis, early cancer, obstructed hernia, and intestinal obstruction unless gangrene or peritonitis has set in. The reaction is of use in the diagnosis more particularly of deep-seated pneumonia, to distinguish gonorrhœal arthritis from ordinary rheumatism, septic or cerebro-spinal meningitis from tuberculous meningitis, cerebral abscess from cerebral tumour, obstructed from strangulated hernia. The relation of the reaction to leucocytosis is that while many of the causes produce both phenomena, there is no necessary connection between the two. In rheumatic fever, for instance, the glycogen reaction is negative though leucocytosis is present; and frequently in leucocytosis-producing diseases the infection is either so slight or so severe that the leucocytes do not rise though the glycogen reaction is positive. One is therefore entitled to regard it as the most delicate test we have of constitutional disturbance in the conditions named. The exact pathological meaning of the reaction is not very clear; it is probably associated with the resistance of the organisms to poisons, and especially so to those which produce positive chemotaxis.

**Leucocytosis and Appendicitis.**—From an examination of 83 consecutive cases of appendicitis in Guy's Hospital French[3] found that leucocytosis alone could not be taken as proof of pus, or of its absence, when it did not exceed 30,000 per cubic millimètre. A leucocyte count of 35,000 or more could be regarded as strong evidence of pus, though lower figures left the question doubtful. More important than the total count was the rising of leucocytosis when the counts were made at intervals, for no such rising occurred in any simple case. The reverse was not true; sometimes pus was present, though there was a progressive diminution in the leucocytosis. Blood-counts must, therefore, be taken into account only in association with the pulse-rate and the general condition of the patient. It is one of several clinical signs, variable like the rest, but often of great value in a difficult case. In discussing this paper, Dudgeon said that a differential count was of great service; in suppuration the polymorphonuclear neutrophiles, which were about 65 per cent., rose to from 80 to 90 per cent. He thought that if there was a leucocyte count of 30,000 leucocytes with 80 per cent. of polymorphonuclears, or 15,000 leucocytes with 90 per cent. of polymorphonuclears, the probability of pus being present was very great.

**Eosinophile Leucocytes.**—From experimental work on guinea-pigs Opie[4] has found that bacteria causing fatal affections diminish the eosinophiles, which gradually disappear from the blood, followed, after a few days should the animal survive, by an increase above the normal. There is thus a contrast and a similarity between the action of animal parasites and bacteria in these cells. Trichinosis, for instance, causes a leucocytosis which is in part due to increase in the eosinophiles and in part to an increase in the ordinary polynuclear cells. The toxins both of the animal parasite and the bacterial toxins are thus chemotactic for the eosinophiles.

**Leukæmia and Sarcomatosis.**—Banti[5] divides leukæmia into lymphatic and myelogenic forms. The lesions in the former may be divided into primary and secondary—those of the liver, serous membranes, lungs, kidneys, and intestines may be looked upon as secondary, and so probably due to hæmatogenous metastases. The newly-formed tissue also invades the neighbouring organs and tissues, and these two facts show that the lymph-adenomatous tissue has a special malignity and behaves as a true neoplasm. In certain sarcomas the same vascular changes are found, the blood circulating in canals without endothelium, and there is also in sarcomas an increase of mono-nuclear cells in the blood more or less like lymphocytes. Between the typical lymphatic leukæmia in which the glands remain distinct and those in which the glands are fused and the organs invaded there are all intermediate forms.

Again, in chloroma there is sometimes a lymph-adenomatous texture, sometimes a sarcomatous round-celled one, and with this a leukæmic increase in the mononuclear cells. Lymphatic leukæmia is therefore a lymph-adenoid sarcomatosis—the sarcomatous tissue invades the vessel walls, destroys the epithelium, and the neoplastic elements penetrate in variable quantities the blood. In myelogenic leukæmia when the lymphatic glands and spleen participate it is found that the lymphatic tissue is replaced by a myeloid tissue reproducing that of the medulla of the bones. This myeloid tissue wherever found shows a more or less atypical structure when compared with the normal medulla, it invades the vessel walls, particularly that of the veins, and destroying the epithelium gets into direct contact with the blood. The cells of the organs in which the metastases are do not take part in the formation of the secondary nodules. Banti has not been able to find any infective agent but believes that the diseases belong to the group of infective diseases whether the specific agent be animal or vegetable.

[1] Brit. Med. Jour., Aug. 13. [2] Ibid, April 16. [3] Lancet May 14. [4] Med. Rec., May 14. [5] Med. Chron, May.

